# Cancer-Related Alopecia Risk and Treatment

**DOI:** 10.1007/s11864-025-01336-2

**Published:** 2025-07-14

**Authors:** Lily Kaufman, Lilia Valentic, Hannah Moulton, Lucy Rose, Brittany Dulmage

**Affiliations:** 1 The Ohio State University College of Medicine, Columbus, OH USA; 2 Department of Dermatology, The Ohio State University Wexner Medical Center, Columbus, OH USA

**Keywords:** Cancer-related alopecia, Chemotherapy-induced hair loss, Endocrine therapy-induced alopecia, Hair regrowth treatments, Scalp cooling therapy, Supportive oncology care

## Abstract

Cancer-related alopecia (CRA) presents a significant challenge for many patients undergoing cancer treatment, often affecting their psychological well-being and sense of identity. In my opinion, the optimal management of CRA requires a proactive, personalized approach that prioritizes both prevention and regrowth, while taking into account the type of cancer therapy, patient goals, and overall clinical context. For patients receiving chemotherapy, especially taxane- or anthracycline-based regimens, scalp cooling should be offered as a first-line preventative option whenever feasible. Its demonstrated effectiveness, particularly when appropriately sequenced with chemotherapy agents, makes it a valuable tool in preserving hair and quality of life. For patients with contraindications to scalp cooling or limited access to this intervention, early counseling and support around hair loss expectations and coping strategies remain critical. In terms of regrowth, topical minoxidil remains the most evidence-based pharmacologic option and should be recommended, especially for patients with endocrine therapy- or chemotherapy-induced alopecia. While oral minoxidil shows promise, it should be used with caution until more robust safety data are available in oncology settings. Spironolactone, tretinoin, prostaglandin analogs, and red light therapy may be considered in select cases, especially when standard options are insufficient, though patients should be counseled on the limitations of available evidence. Ultimately, a patient-centered, multidisciplinary approach is key to optimizing outcomes in CRA care.

## Introduction

Cancer-related alopecia (CRA) is a common and distressing side effect of cancer treatment. Quality of life is a significant challenge for patients experiencing CRA, as it can impact mental health, self-esteem, body image, sexuality, and social functioning [1]. Given these challenges, it is important to explore strategies for managing CRA with consideration of how the risk of CRA varies by treatment regimen. Tailored approaches to CRA management are essential to address patients’ unique needs. This article summarizes the impact of different anti-cancer drug classes on hair loss and discusses treatment options for prevention and regrowth.

## Cancer Treatments and Alopecia Risk

Cancer treatments vary widely in their mechanisms of action, and this diversity extends to their impact on hair follicles. The risk, pattern, and permanence of alopecia depend on the specific drug class, dosage, and duration of treatment. Knowledge of the hair growth cycle may aid in the understanding of mechanisms by which specific treatments contribute to alopecia. The hair cycle consists of three stages with varying growth patterns. The anagen phase is marked by active division and regeneration of hair follicles, leading to hair growth. This phase is believed to last between one and six years with variation from person to person, with 80–90% of hair follicles in anagen phase at any given time. The next phase is the catagen phase, which is characterized by separation of the hair follicle and shaft via apoptosis-driven involution. This phase is brief, and is believed to last for several weeks and involve less than one percent of hair follicles at a given time. Finally, during the telogen phase, often thought of as the “resting” phase, the hair is in a quiescent state prior to leaving the hair shaft. This phase is believed to last between three and nine months, with 10–20% of hair follicles in this state at a given time. Following this phase, the hair sheds and the cycle repeats [2]. The following sections categorize cancer treatments by drug class and summarize their associated risk and pattern of alopecia, highlighting key differences across treatment types.

### Chemotherapy-Induced Alopecia

Chemotherapy-induced alopecia (CIA) impacts a significant proportion of patients undergoing cancer treatment with chemotherapy, with an estimated incidence of 65% [3]. Chemotherapy is used in the treatment of a wide range of cancers, including solid tumors as well as hematologic malignancies. The risk, pattern, and severity of CIA varies by treatment type and protocol, with certain drug classes carrying a particularly high likelihood of hair loss.

Taxanes such as paclitaxel and docetaxel are used to treat a variety of cancers including endometrial cancer, non-small-cell lung cancer, breast cancer, bladder cancer, cervical carcinoma [4], head and neck malignancies, prostate cancer, and gastric adenocarcinoma [5]. Taxanes are among the leading causes of CIA, with a high likelihood of causing extensive, and in some cases permanent, hair loss [6]. The frequency of CIA has been reported to exceed 80% in patients treated with taxane therapy [3], with at least 60% of patients experiencing diffuse grade 2 alopecia of the scalp [6]. With cumulative therapy, the risk and severity of hair loss increases, often leading to loss of body hair including the eyebrows, eyelashes, beard, pubic and axillary hair, and other body hair [7]. Hair loss typically occurs via a mechanism of chemotherapy-induced damage to hair follicles in the anagen phase, inducing dystrophic anagen effluvium. Mechanisms of telogen effluvium have also been described [3]. Hair loss is typically reversible, with gradual hair regrowth beginning three to six months after the final chemotherapy cycle. However, changes in the baseline texture or color of the regrown hair are common [8]. Less frequently, persistent chemotherapy-induced alopecia (pCIA) has been used to describe suboptimal or absent hair regrowth on the scalp and body more than six months after treatment discontinuation [9]. pCIA hair loss is not total and is typically diffuse or accentuated in areas prone to androgenetic alopecia, and is characterized by finer, shorter hairs with a healthy-appearing scalp. There are no long-term studies to confirm the perceived permanence of pCIA [6].

Anthracyclines such as doxorubicin and daunorubicin, used to treat a variety of malignancies including leukemias, lymphomas, sarcomas, and bladder, breast, gynecological, and other metastatic cancers [10], represent another class of chemotherapy drugs commonly associated with CIA. Alopecia has been reported in nearly all patients receiving anthracycline-containing regimens at a rate of 60–100% [11]. Alkylating agents such as cyclophosphamide, frequently used to treat breast cancer, leukemia, lymphoma, multiple myeloma, neuroblastoma, retinoblastoma, and ovarian cancer [12], are associated with an alopecia incidence of 60% or higher [3]. In contrast, antimetabolites such as 5-fluorouracil and capecitabine, used in the treatment of breast, esophageal, laryngeal, gastrointestinal, and genitourinary neoplasms [13], have a reported alopecia incidence between 10 and 50%. Similarly to taxanes, these chemotherapy drugs are associated with diffuse, gradual or rapid scalp shedding beginning one to three weeks after the initiation of chemotherapy, with near complete loss of all scalp and body hair resulting from cumulative treatment. Alopecia is typically reversible, with regrowth of hair beginning three to six months after termination of therapy [3]. Polychemotherapy regimens are associated with a higher incidence CIA relative to monotherapy [14]. Chemotherapy drugs and their relative risks and patterns of hair loss are summarized in Table 1.

**Table 1:** Alopecia risk by cancer and treatment type

Drug Class	Cancers Treated	Alopecia Risk	Pattern of Hair Loss
Chemotherapy			
Taxanes (paclitaxel, docetaxel)	Endometrial, non-small-cell lung, breast, bladder, cervical [4], head and neck, prostate, gastric [5]	60–80% or higher [3, 6]	Diffuse grade 2 scalp alopecia [6] Increased severity with cumulative therapy to include eyebrows, eyelashes, beard, pubic and axillary hair, and other body hair [7] Potential for pCIA with diffuse suboptimal or absent hair growth accentuated in areas prone to androgenetic alopecia [6, 9]
Anthracyclines (doxorubicin, daunorubicin)	Leukemia, lymphoma, sarcoma, bladder, breast, gynecological, metastatic disease [10]	60–100% [11]	Diffuse, gradual, or rapid scalp shedding beginning one to three weeks after initiation of chemotherapy Near complete loss of all scalp and body hair with cumulative treatment [3]
Alkylating agents (cyclophosphamide)	Breast, leukemia, lymphoma, multiple myeloma, neuroblastoma, retinoblastoma, ovarian [12]	60% or higher [3]	Diffuse, gradual, or rapid scalp shedding beginning one to three weeks after initiation of chemotherapy Near complete loss of all scalp and body hair with cumulative treatment [3]
Antimetabolites (capecitabine, 5-fluorouracil)	Breast, esophageal, laryngeal, gastrointestinal, genitourinary [13]	10–50% [3]	Diffuse, gradual, or rapid scalp shedding beginning one to three weeks after initiation of chemotherapy Near complete loss of all scalp and body hair with cumulative treatment with 5-fluorouracil [3]
Endocrine Therapy			
Selective estrogen receptor modulators (tamoxifen), Aromatase inhibitors (anastrozole)	Breast, prostate, ovarian, endometrial [15, 16]	~ 25% [16]	Gradual, diffuse hair thinning of the frontal and bitemporal scalp Complete baldness rare Resembles androgenetic alopecia [16]
CDK 4/6 Inhibitors			
Palbociclib, ribociclib, abemaciclib	Frequently combined with endocrine therapies to treat cancers including leukemia, breast, lung, head, neck, cervical, prostate, neuroblastoma [20]	15–30% [18, 19]	Androgenetic pattern [15, 18, 23] More severe vertex involvement in combination with endocrine therapy [24]
Targeted Therapies and Antibody–Drug Conjugates			
EGFR inhibitors (small-molecule inhibitors erlotinib and gefitinib, mAbs cetuximab and panitumumab)	Breast, colorectal pancreatic, lung, leukemia, lymphoma, multiple myeloma [26]	< 10% [28–30]	Androgenetic [31], frontal [32], or diffuse [12] pattern most common Cicatricial alopecia [33], folliculitis decalvans [34], and inflammatory nonscarring [35] and scarring alopecia have been observed Scalp alopecia accompanied by hirsutism and eyebrow and eyelash trichomegaly [12]
MEK inhibitors (trametinib, binimetinib)	Breast, colorectal pancreatic, lung, leukemia, lymphoma, multiple myeloma [26]	17% [37]	Grade I diffuse thinning most common [37] Inflammatory alopecia has been observed [38]
HER2-targeted therapies (trastuzumab, pertuzumab)	HER2-positive gastric, biliary, colorectal, non-small-cell lung, bladder [39]	Generally not associated with alopecia [41]	N/A
HER2-antibody–drug conjugates (trastuzumab deruxtecan, trastuzumab emtansine)	HER2-positive gastric, biliary, colorectal, non-small-cell lung, bladder [39]	36–48% for trastuzumab deruxtecan, 3% for trastuzumab emtansine [42–44]	Typically grade I diffuse [42–44]
Trop-2-antibody–drug conjugate (sacituzumab govitecan)	Metastatic triple negative breast cancer [45, 46]	46–76% [45, 46]	Typically grade I diffuse [45, 46]
Checkpoint Inhibitors			
CTLA-4 inhibitor (ipilimumab)	Metastatic melanoma and other solid organ malignancies [47]	Rare, limited to a few case reports [48–50]	Alopecia areata and alopecia universalis, treatment-concurrent or remote onset [48–50]
PD-1 receptor inhibitors (pembrolizumab, nivolumab, cemiplimab) and PD-L1 receptor inhibitors (atezolizumab, durvalumab)	Metastatic melanoma and other solid organ malignancies [47]	1–2% [51]	Alopecia areata [51] Patchy or diffuse hair loss involving the scalp, eyebrows, beard, or universal hair loss beginning after several months of treatment [52, 53] Poliosis of regrown hair is typical [54] Persistent hair loss has been observed in up to 31% of cases [55]

*pCIA*, persistent chemotherapy-induced alopecia; *CDK*, cyclin-dependent kinase; *EGFR*, epidermal growth factor receptor; *mAb*, monoclonal antibody; *MEK*, mitogen-activated protein kinase; *HER2*, human epidermal growth factor receptor 2; *Trop-2*, human trophoblast cell-surface antigen 2; *CTLA-4*, cytotoxic T lymphocyte-associated antigen-4; *PD-1*, programmed cell death protein 1; *PD-L1*, programmed cell death ligand 1

### Endocrine Therapy-Induced Alopecia

Endocrine therapy-induced alopecia (EIA) is a recognized side effect of hormone-targeting treatments used to treat breast, prostate, ovarian, and endometrial cancers [15, 16]. Unlike CIA, EIA typically presents as gradual, diffuse hair thinning rather than abrupt shedding, and rarely leads to complete baldness. The risk of alopecia varies depending on the specific therapy, with an overall all-grade alopecia incidence of 4.4%. Selective estrogen receptor modulators such as tamoxifen has been reported to have the highest incidence of alopecia at 25.4%, followed by aromatase inhibitors such as anastrozole at 25%. Studies have not consistently found a link between androgen deprivation therapy and alopecia, though research on this association remains limited [16]. The pattern of hair loss in EIA commonly resembles androgenetic alopecia, with progressive thinning at the frontal scalp and crown beginning within three to six months after starting treatment [12, 15]. The mechanism of EIA is believed to be similar to that of androgenetic alopecia, and is characterized by a reduction in the action of estrogen on the hair follicle leading to a gradual shortening of the anagen phase resulting in follicle miniaturization and subsequent fragility, breakage, and hair loss [16]. Regrowth after treatment discontinuation varies, with initial improvement often seen within three to six months, though full regrowth may take a year or longer, and some patients report persistent hair thinning [17].

### Cyclin-Dependent Kinase (CDK) 4/6 Inhibitors and Alopecia

Treatment with CDK 4/6 inhibitors such as palbociclib, ribociclib, and abemaciclib has been associated with the progressive development of non-scarring alopecia in 15–30% of exposed patients, [18, 19], with a median onset time of 67 days. CDK 4/6 inhibitors are commonly used in combination with endocrine therapies to treat leukemia, breast, lung, head, neck, cervical, and prostate cancers, and neuroblastomas [20], and studies suggest that this combined approach may further heighten the risk of alopecia compared to endocrine therapy alone, with an estimated pooled incidence of 23% compared to 9.6% of patients treated with endocrine monotherapy [18] [19, 21, 22]. Alopecia due to CDK 4/6 inhibitor therapy is typically grade I and occurs in an androgenetic pattern [15, 18, 23], with more severe vertex involvement observed among patients taking combination endocrine and CDK 4/6 inhibitor therapy. Eyebrow and eyelash thinning has also been observed. Although CDK 4/6 inhibitor-induced alopecia is generally considered to be temporary, long-term outcomes are not well studied, and prolonged treatment may contribute to delayed or persistent hair thinning in some patients [24]. The mechanism of CDK 4/6 inhibitor related alopecia is believed to be similar to that of androgenetic and endocrine therapy-induced alopecia, although further studies are required to characterize the exact mechanism of hair loss [25].

### Targeted Therapies and Antibody–Drug Conjugates

Targeted therapies, comprised of small molecule inhibitors and monoclonal antibodies (mAbs), have been used to treat a variety of hematologic and solid malignancies including breast, colorectal, pancreatic, and lung cancer, as well as leukemia, lymphoma, and multiple myeloma [26]. One major class of targeted therapy is epidermal growth factor receptor (EGFR) inhibitors, which include both small-molecule inhibitors (erlotinib, gefitinib) and mAbs (cetuximab, panitumumab) [27]. EGFR inhibitors are associated with a less than 10% incidence [28–30] of mild hair loss in an androgenetic [31], frontal [32], or diffuse [12] pattern, although cicatricial alopecia [33], folliculitis decalvans [34], and inflammatory nonscarring [35] and scarring alopecia have also been observed. Scalp alopecia is accompanied by hirsutism and eyebrow and eyelash trichomegaly [12]. The mechanism of scalp and body hair loss can be explained by EGFR inhibitors disrupting follicular cycling and increasing oxidative stress, while paradoxically stimulating eyebrow, eyelash, and facial hair growth due to differing dependencies on EGFR signaling [32].

In addition to EGFR inhibitors, other small-molecule targeted therapies have been associated with alopecia. Mitogen-activated protein kinase (MEK) inhibitors such as trametinib and binimetinib [36] have been reported to cause alopecia in approximately 17% of patients, typically presenting as grade I diffuse thinning [37], although inflammatory alopecia has also been observed [38]. CDK 4/6 inhibitors also fall within the small-molecule inhibitor category of targeted therapy, but have been discussed separately in this manuscript due to their distinct pattern of hair loss [15, 18, 23, 24].

Additional examples of mAbs associated with alopecia include human epidermal growth factor receptor 2 (HER2)-targeted therapies such as trastuzumab and pertuzumab [39] and antibody–drug conjugates (ADCs) [40]. HER2-targeted therapies and ADCs are used to treat HER2-positive breast cancers and other HER2-positive solid tumors, including gastric, biliary, colorectal, non-small-cell lung, and bladder cancers [39]. Trastuzumab and pertuzumab are generally not associated with alopecia [41]. HER2 ADCs, however, are associated with alopecia due to their linkage to a cytotoxic payload [40]. Alopecia has been reported in 36–48% of patients treated with trastuzumab deruxtecan and 3% of patients treated with trastuzumab emtansine, typically presenting as grade I [42–44]. Sacituzumab govitecan is an ADC targeting the human trophoblast cell-surface antigen 2 (Trop-2) that is frequently used to treat metastatic triple negative breast cancer. Sacituzumab govitecan is associated with an estimated alopecia incidence between 46–76% that is typically grade I [45, 46]. Alopecia due to HER2- and Trop-2 therapies is believed to result from the cytotoxic effects of chemotherapy agents on the rapidly dividing hair shaft cells [8].

### Checkpoint Inhibitors (Immunotherapy-Induced Alopecia)

Immune checkpoint inhibitor therapies are commonly used to treat metastatic melanoma and other solid organ malignancies [47]. The cytotoxic T lymphocyte-associated antigen-4 (CTLA-4) inhibitor ipilimumab has been associated with non-scarring alopecias including alopecia areata and alopecia universalis, with both treatment-concurrent and remote onset of hair loss [48, 49] [50]. Alopecia areata has also been observed in patients undergoing treatment with programmed cell death protein 1 (PD-1) (pembrolizumab) or programmed cell death ligand 1 (PD-L1) (atezolizumab) receptor inhibitors at an incidence rate of 1–2% [51]. Observed hair loss patterns include patchy or diffuse hair loss involving the scalp, eyebrows, and beard, or universal hair loss beginning after several months of treatment [52, 53]. Poliosis of regrown hair is typical [54]. Alopecia has been found to be persistent in some cases, with reports of no regrowth at 12 months as high as 31% in a case series. The mechanism of immunotherapy-induced alopecia can be understood as a manifestation of autoimmunity induced by checkpoint inhibitor therapy [55].

## Treatment Strategies for Cancer-Related Alopecia

### Preventative Measures

Scalp cooling (SC) caps work by reducing blood flow to the scalp through cold-induced vasoconstriction, limiting the delivery of chemotherapeutic agents to hair follicles and reducing their cellular uptake [14]. These devices come in various forms, including mechanically controlled, tight-fitting caps that circulate cooled gel or fluid, as well as manually frozen or refrigerated caps. The most used automated FDA-approved SC caps include the DigniCap, Paxman, and Amma brands [56]. These systems use continuous cooling and precise temperature regulation to enhance effectiveness. Studies have demonstrated that SC significantly reduces chemotherapy-induced alopecia (CIA) when the cap is applied at temperatures below 0 °C for at least 30 minutes prior to infusion and maintained for up to 90 minutes post-infusion [57]. The greatest efficacy has been observed in patients receiving taxane- or anthracycline-based chemotherapy regimens [3, 57]. While scalp cooling offers a non-invasive and potentially cost-effective option for hair preservation, it is contraindicated in patients with certain hematologic malignancies due to concerns about reduced chemotherapy efficacy in the scalp region [14, 57]. Reported side effects are generally mild, with headaches and cold-induced discomfort being the most common, and no serious adverse effects documented [3, 14].

Recent findings further support the effectiveness of SC in preventing chemotherapy-induced alopecia, particularly with taxane-based regimens. SC was most successful in patients receiving paclitaxel-only chemotherapy (100% hair retention) and docetaxel-containing non-anthracycline regimens (88%). Notably, chemotherapy sequencing influenced SC success rates, with patients receiving paclitaxel before anthracyclines achieving higher rates of hair retention (73%) compared to those receiving anthracyclines first (44%). These findings suggest that prolonged scalp hypothermia during the taxane phase may enhance follicular protection against subsequent anthracycline-induced damage. SC was less effective in dose-dense regimens, with only 60% of patients achieving satisfactory hair retention. Most SC failures occurred within the first few treatment cycles, but some patients experienced hair loss later in their regimen, indicating no predictable pattern of alopecia onset. Overall, SC was well tolerated, with only 4% of patients discontinuing due to discomfort, and no serious adverse events reported. These results highlight the potential for chemotherapy sequencing to optimize SC efficacy, which may influence treatment decisions in patients prioritizing hair preservation [58].

### Hair Regrowth After Cancer Treatment

#### FDA-Approved Treatments

Topical minoxidil, one FDA-approved pharmacotherapy for pattern hair loss, acts as an arteriolar vasodilator [59, 60]. By opening potassium channels, minoxidil may induce vascular growth factor expression, stimulate microcirculation around hair follicles, and activate the prostaglandin-endoperoxide synthase-1 enzyme, all of which can lead to enhanced hair growth. Minoxidil is available in both topical and oral forms as a treatment for CRA [60]. 2% topical minoxidil was shown to decrease the duration of alopecia induced by chemotherapy in a randomized double-blind trial. This study showed that the participants who used minoxidil had a longer time interval until maximum hair loss occurred compared to those who used placebo [61]. 5% topical minoxidil has also demonstrated beneficial effects on hair regrowth in post chemotherapy alopecia [15, 62]. A single-blind study found that 80% of breast cancer patients who used 5% topical minoxidil showed a moderate to significant improvement in alopecia among patients with EIA [15]. The efficacy of oral minoxidil to treat CRA is an emerging area of study, with less data supporting its efficacy. Case reports have reported significant improvement in hair regrowth and an increased number of growing follicles in patients with pCIA treated with oral minoxidil [63, 64]. Cohort studies have reported similar findings, with one study demonstrating greater benefit when oral minoxidil was combined with topical minoxidil treatment compared to topical monotherapy in patients with pCIA and EIA [65]. A retrospective study comparing oral and topical minoxidil found similar treatment response rates in patients with EIA, while oral minoxidil was superior for patients with pCIA [66].

Spironolactone is another commonly used treatment for CRA. Spironolactone is a synthetic aldosterone receptor antagonist, blocking androgens and reducing the effects of testosterone in women. Spironolactone has been shown to have beneficial effects on hair growth in patients with androgenetic alopecia [67–69]. However, there is a lack of data on the effect of spironolactone on hormone-sensitive hair loss post-endocrine therapy specifically. In recent studies, spironolactone is often used in conjunction with topical minoxidil, making it difficult to understand the effects of spironolactone alone on hair growth. A retrospective study found that patients with both persistent chemotherapy-induced alopecia and endocrine therapy-induced alopecia showed significant to moderate improvement in hair growth [62]. Although spironolactone has been shown to have beneficial effects in androgenetic alopecia and female pattern hair loss, the gap in the literature regarding the sole effects of spironolactone on CRA indicates the need for further research. Additionally, more long-term data is needed to elucidate the safety profile of spironolactone in the setting of hormone-receptor positive cancers.

### Emerging and Investigational Treatments

Recent studies have investigated the potential of topical tretinoin to enhance the efficacy of minoxidil in treating androgenetic alopecia by upregulating follicular sulfotransferase enzymes. Researchers found that applying topical tretinoin influenced the expression of these enzymes, which are critical for minoxidil’s activation within hair follicles. Notably, 43% of participants who were initially predicted to be unresponsive to minoxidil experienced a positive response after five days of tretinoin application. This suggests that combining topical tretinoin with minoxidil may improve treatment outcomes for patients with androgenetic alopecia [70].

Prostaglandins play a key role in regulating the hair growth cycle and have been found to stimulate eyelash growth by prolonging the anagen (growth) phase, and both prescription and over-the-counter lash serums containing prostaglandins have been promoted to support eyelash growth. These compounds enhance eyelash length, thickness, and darkness by prolonging the anagen (growth) phase and reducing the latency period between the telogen (resting) and anagen phases. Bimatoprost, an FDA-approved prostaglandin analog, has demonstrated significant efficacy in clinical settings [71, 72]. However, the use of prostaglandin analogs can lead to side effects such as periocular skin hyperpigmentation and, in rare cases, changes in iris color [73]. Therefore, it is essential for both patients and healthcare providers to weigh the benefits and potential risks when considering these treatments for eyelash enhancement [74].

Red light therapy (RLT), also known as photobiomodulation therapy, has been recently studied as a treatment for chemotherapy-induced alopecia. A randomized controlled study of RLT in cancer patients demonstrated that RLT significantly accelerated hair growth post-chemotherapy, with participants receiving three RLT sessions per week over 12 weeks showing improved hair density and quality of life compared to controls [75]. Further studies are required to establish standardized guidelines and recommendations for RLT use as a treatment for chemotherapy-induced alopecia.

### Supplements

Due to the limitations of conventional alopecia treatments, many patients seek complementary and alternative medicine options, including natural products and supplements [67]. Various compounds, particularly biotin and specific hair growth supplements, have been explored for regrowth, although research has largely focused on patients with androgenetic alopecia and alopecia areata rather than patients experiencing cancer-related alopecia [76, 77]. Although biotin has been popularized as a treatment for hair loss, there are no randomized controlled trials to support its efficacy for treating alopecia of any kind [77]. Nutraceuticals such as Nutrafol and Viviscal have been studied for their efficacy in treating hair loss. Several trials have demonstrated the ability of Viviscal to promote terminal and vellus hair growth, decrease hair loss, and increase hair shaft diameter [78–80]. Additionally, several studies have supported Nutrafol’s ability to promote increased hair growth and improved hair quality [81, 82]. A summary of treatment methods for cancer-related alopecia are summarized in Table 2.

**Table 2:** Cancer-related alopecia prevention and treatment methods

Treatment	Strength of Evidence	Notes
Scalp cooling therapy	Highly effective in taxane-based regimens, especially paclitaxel-only (100% hair retention) and docetaxel without anthracyclines (88% hair retention) with better outcomes when taxanes precede anthracyclines [58]	Contraindicated in patients with certain hematologic malignancies due to concerns about reduced chemotherapy efficacy in the scalp region [14, 57]
Topical and oral minoxidil	Moderate evidence supports topical minoxidil for reducing chemotherapy-induced alopecia duration and promoting regrowth, particularly in endocrine therapy induced alopecia [15, 61, 62] Emerging evidence for oral minoxidil, with case reports and small cohort studies suggesting benefit in pCIA [63–66]	Combination oral and topical minoxidil therapy may improve outcomes over topical monotherapy [65]
Spironolactone	Limited evidence for spironolactone in CRA, retrospective studies show improvement in androgenetic alopecia when used with topical minoxidil [67–69] Retrospective study evaluating efficacy for pCIA and endocrine therapy induced alopecia found significant to moderate improvement in hair growth [62]	Commonly used in androgenetic and female pattern hair loss [67–69] Commonly combined with topical minoxidil [62]
Topical tretinoin	Preliminary evidence from short-term studies suggest topical tretinoin may improve minoxidil responsiveness in androgenetic alopecia [70]	No studies evaluating combination tretinoin/minoxidil effectiveness for treating alopecia resulting from cancer therapies
Prostaglandin analogs	Strong evidence supports the efficacy of prostaglandin analogues for enhancing eyelash growth [71, 72]	Bimatoprost FDA-approved [71, 72] Potential side effects of periocular skin hyperpigmentation and changes in iris color [73]
Red light therapy	Preliminary evidence suggests that red light therapy may improve post-chemotherapy hair regrowth and quality of life [75]	No standardized treatment protocols
Supplements	Limited evidence for natural products in cancer-related alopecia, some trials support Viviscal and Nutrafol for hair growth in androgenetic alopecia, but no randomized controlled trials support biotin’s efficacy for any form of alopecia [76–82]	Research does not evaluate efficacy for cancer related alopecia

*pCIA*, persistent chemotherapy-induced alopecia; *FDA*, Food and Drug Administration

## Conclusion

Cancer-related alopecia is a distressing side effect of cancer treatment that significantly impacts patients’ quality of life. The risk, pattern, and severity of alopecia vary by drug class, with chemotherapy, endocrine therapies, targeted agents, and immunotherapies each contributing to hair loss through distinct mechanisms. While scalp cooling remains the most well-established preventative measure, emerging treatment options, including minoxidil, spironolactone, red light therapy, and prostaglandin analogs, show promise in promoting regrowth. Nutritional supplements such as biotin and nutraceuticals are widely used, though evidence supporting their efficacy remains limited. Further research is needed to refine treatment strategies and develop targeted interventions to mitigate hair loss and improve outcomes for patients experiencing cancer-related alopecia.

## Data Availability

No datasets were generated or analysed during the current study.

## References

[1] Lemieux J, Maunsell E, Provencher L. Chemotherapy-induced alopecia and effects on quality of life among women with breast cancer: a literature review. Psychooncology. 2008;17(4):317–28. 10.1002/pon.1245. 17721909 10.1002/pon.1245

[2] Paus R. Principles of hair cycle control. J Dermatol. 1998;25(12):793–802. 10.1111/j.1346-8138.1998.tb02507.x. 9990771 10.1111/j.1346-8138.1998.tb02507.x

[3] Trüeb RM. Chemotherapy-induced hair loss. Skin Therapy Lett. 2010;15(7):5–7. 20700552

[4] Alqahtani FY, Aleanizy FS, El Tahir E, Alkahtani HM, AlQuadeib BT. Paclitaxel. Profil Drug Subst Excip Relat Methodol. 2019;44:205–38. 10.1016/bs.podrm.2018.11.001. 10.1016/bs.podrm.2018.11.001 31029218

[5] Gupta R, Kadhim MM, Turki Jalil A, Qasim Alasheqi M, Alsaikhan F, Khalimovna Mukhamedova N, et al. The interactions of docetaxel with tumor microenvironment. Int Immunopharmacol. 2023;119: 110214. 10.1016/j.intimp.2023.110214. (Highlights evolving understanding of taxane-chemotherapy interaction with the tumor microenvironment, contributing to mechanistic insights into hair follicle vulnerability). 37126985 10.1016/j.intimp.2023.110214

[6] Sibaud V, Lebœuf NR, Roche H, et al. Dermatological adverse events with taxane chemotherapy. Eur J Dermatol. 2016;26(5):427–43. 10.1684/ejd.2016.2833. 27550571 10.1684/ejd.2016.2833 PMC5526115

[7] Rowinsky EK, Donehower RC. Paclitaxel (taxol). N Engl J Med. 1995;332(15):1004. 10.1056/NEJM199504133321507. 7885406 10.1056/NEJM199504133321507

[8] Paus R, Haslam IS, Sharov AA, Botchkarev VA. Pathobiology of chemotherapy-induced hair loss. Lancet Oncol. 2013;14(2):e50–9. 10.1016/S1470-2045(12)70553-3. 23369683 10.1016/S1470-2045(12)70553-3

[9] Tallon B, Blanchard E, Goldberg LJ. Permanent chemotherapy-induced alopecia: case report and review of the literature. J Am Acad Dermatol. 2010;63(2):333–6. 10.1016/j.jaad.2009.06.063. 20471136 10.1016/j.jaad.2009.06.063

[10] Cejas RB, Petrykey K, Sapkota Y, Burridge PW. Anthracycline toxicity: light at the end of the tunnel? Annu Rev Pharmacol Toxicol. 2024;64:115–34. 10.1146/annurev-pharmtox-022823-035521. (Provides a contemporary review of anthracycline toxicity, including mechanisms relevant to hair follicle damage. Useful for contextualizing alopecia within broader toxicity). 37788492 10.1146/annurev-pharmtox-022823-035521 PMC11260185

[11] Jasra S, Anampa J. Anthracycline use for early stage breast cancer in the modern era: a review. Curr Treat Options Oncol. 2018;19(6): 30. 10.1007/s11864-018-0547-8. 29752560 10.1007/s11864-018-0547-8

[12] Freites-Martinez A, Shapiro J, Goldfarb S, et al. Hair disorders in patients with cancer. J Am Acad Dermatol. 2019;80(5):1179–96. 10.1016/j.jaad.2018.03.055. 29660422 10.1016/j.jaad.2018.03.055 PMC6186204

[13] Walko CM, Lindley C. Capecitabine: a review. Clin Ther. 2005;27(1):23–44. 10.1016/j.clinthera.2005.01.005. 15763604 10.1016/j.clinthera.2005.01.005

[14] Rossi A, Fortuna MC, Caro G, et al. Chemotherapy-induced alopecia management: clinical experience and practical advice. J Cosmet Dermatol. 2017;16(4):537–41. 10.1111/jocd.12308. 28150447 10.1111/jocd.12308 PMC5540831

[15] Freites-Martinez A, Shapiro J, Chan D, et al. Endocrine therapy-induced alopecia in patients with breast cancer. JAMA Dermatol. 2018;154(6):670–5. 10.1001/jamadermatol.2018.0454. 29641806 10.1001/jamadermatol.2018.0454 PMC6145643

[16] Saggar V, Wu S, Dickler MN, Lacouture ME. Alopecia with endocrine therapies in patients with cancer. Oncologist. 2013;18(10):1126–34. 10.1634/theoncologist.2013-0193. 24037977 10.1634/theoncologist.2013-0193 PMC3805155

[17] Kuo AM, Reingold RE, Ketosugbo KF, et al. Oral minoxidil for late alopecia in cancer survivors. Breast Cancer Res Treat. 2024;208(3):491–9. 10.1007/s10549-024-07440-5. (One of few studies exploring oral minoxidil in cancer survivors with persistent alopecia, offering evidence for regrowth where data is otherwise limited). 39097564 10.1007/s10549-024-07440-5

[18] Sibaud V, Sollena P. Dermatologic toxicities to inhibitors of cyclin-dependent kinases CDK 4 and 6: an updated review for clinical practice. Ann Dermatol Venereol. 2023;150(3):208–12. 10.1016/j.annder.2022.11.013. 37586898 10.1016/j.annder.2022.11.013

[19] Turner NC, Ro J, André F, et al. Palbociclib in hormone-receptor-positive advanced breast cancer. N Engl J Med. 2015;373(3):209–19. 10.1056/NEJMoa1505270. (Summarizes updated clinical findings in CDK 4/6 inhibitor-induced skin toxicities, including alopecia—an important emerging side effect in targeted therapie**s**). 26030518 10.1056/NEJMoa1505270

[20] Hassan MA, Ates-Alagoz Z. Cyclin-dependent kinase 4/6 inhibitors against breast cancer. Mini Rev Med Chem. 2023;23(4):412–28. 10.2174/1389557522666220606095540. (Describes CDK 4/6 inhibitors in oncology and supports mechanistic understanding of hair loss related to these agents). 35670349 10.2174/1389557522666220606095540

[21] Tripathy D, Im SA, Colleoni M, et al. Ribociclib plus endocrine therapy for premenopausal women with hormone-receptor-positive, advanced breast cancer (MONALEESA-7): a randomised phase 3 trial. Lancet Oncol. 2018;19(7):904–15. 10.1016/S1470-2045(18)30292-4. 29804902 10.1016/S1470-2045(18)30292-4

[22] Finn RS, Martin M, Rugo HS, et al. Palbociclib and letrozole in advanced breast cancer. N Engl J Med. 2016;375(20):1925–36. 10.1056/NEJMoa1607303. 27959613 10.1056/NEJMoa1607303

[23] Raschi E, Fusaroli M, La Placa M, et al. Skin toxicities with cyclin-dependent kinase 4/6 inhibitors in breast cancer: signals from disproportionality analysis of the FDA adverse event reporting system. Am J Clin Dermatol. 2022;23(2):247–55. 10.1007/s40257-021-00645-0. (Uses FDA safety data to identify signal strength for CDK 4/6 inhibitor-induced alopecia, helping quantify real-world incidence.). 34699032 10.1007/s40257-021-00645-0

[24] Minta A, Rose L, Park C, et al. Retrospective cohort study of CDK4/6-inhibitor-induced alopecia in breast cancer patients. Support Care Cancer. 2023;31(12): 717. 10.1007/s00520-023-08160-0. (One of the first retrospective cohorts to evaluate alopecia outcomes in CDK 4/6 therapy, providing practice-relevant epidemiologic data). 37991653 10.1007/s00520-023-08160-0

[25] Silvestri M, Cristaudo A, Morrone A, et al. Emerging skin toxicities in patients with breast cancer treated with new cyclin-dependent kinase 4/6 inhibitors: a systematic review. Drug Saf. 2021;44(7):725–32. 10.1007/s40264-021-01071-1. 33959899 10.1007/s40264-021-01071-1

[26] Gerber DE. Targeted therapies: a new generation of cancer treatments. Am Fam Physician. 2008;77(3):311–9. 18297955

[27] Macdonald JB, Macdonald B, Golitz LE, LoRusso P, Sekulic A. Cutaneous adverse effects of targeted therapies: Part I: inhibitors of the cellular membrane. J Am Acad Dermatol. 2015;72(2):203–20. 10.1016/j.jaad.2014.07.032. 25592338 10.1016/j.jaad.2014.07.032

[28] Lilenbaum R, Axelrod R, Thomas S, et al. Randomized phase II trial of erlotinib or standard chemotherapy in patients with advanced non-small-cell lung cancer and a performance status of 2. J Clin Oncol. 2008;26(6):863–9. 10.1200/JCO.2007.13.2720. 18281658 10.1200/JCO.2007.13.2720

[29] Belum VR, Marulanda K, Ensslin C, et al. Alopecia in patients treated with molecularly targeted anticancer therapies. Ann Oncol. 2015;26(12):2496–502. 10.1093/annonc/mdv390. 26387145 10.1093/annonc/mdv390 PMC4658542

[30] Ciuleanu T, Stelmakh L, Cicenas S, et al. Efficacy and safety of erlotinib versus chemotherapy in second-line treatment of patients with advanced, non-small-cell lung cancer with poor prognosis (TITAN): a randomised multicentre, open-label, phase 3 study. Lancet Oncol. 2012;13(3):300–8. 10.1016/S1470-2045(11)70385-0. 22277837 10.1016/S1470-2045(11)70385-0

[31] Segaert S, Chiritescu G, Lemmens L, Dumon K, Van Cutsem E, Tejpar S. Skin toxicities of targeted therapies. Eur J Cancer. 2009;45(Suppl 1):295–308. 10.1016/S0959-8049(09)70044-9. 19775626 10.1016/S0959-8049(09)70044-9

[32] Mounessa J, Caravaglio JV, Domozych R, et al. Commonly prescribed medications associated with alopecia. J Am Acad Dermatol. 2023;88(6):1326-37.e2. 10.1016/j.jaad.2017.01.060. 37268392 10.1016/j.jaad.2017.01.060

[33] Yang BH, Bang CY, Byun JW, et al. A case of cicatricial alopecia associated with erlotinib. Ann Dermatol. 2011;23(Suppl 3):S350–3. 10.5021/ad.2011.23.S3.S350. 22346276 10.5021/ad.2011.23.S3.S350 PMC3276795

[34] Hoekzema R, Drillenburg P. Folliculitis decalvans associated with erlotinib. Clin Exp Dermatol. 2010;35(8):916–8. 10.1111/j.1365-2230.2010.03852.x. 20456379 10.1111/j.1365-2230.2010.03852.x

[35] Pongpudpunth M, Demierre MF, Goldberg LJ. A case report of inflammatory nonscarring alopecia associated with the epidermal growth factor receptor inhibitor erlotinib. J Cutan Pathol. 2009;36(12):1303–7. 10.1111/j.1600-0560.2009.01275.x. 19519875 10.1111/j.1600-0560.2009.01275.x

[36] Macdonald JB, Macdonald B, Golitz LE, LoRusso P, Sekulic A. Cutaneous adverse effects of targeted therapies: Part II: inhibitors of intracellular molecular signaling pathways. J Am Acad Dermatol. 2015;72(2):221–38. 10.1016/j.jaad.2014.07.033. 25592339 10.1016/j.jaad.2014.07.033

[37] Flaherty KT, Robert C, Hersey P, et al. Improved survival with MEK inhibition in BRAF-mutated melanoma. N Engl J Med. 2012;367(2):107–14. 10.1056/NEJMoa1203421. 22663011 10.1056/NEJMoa1203421

[38] Rose L, Minta A, Plaza J, Cohn DE, Dulmage B. Mitogen-activated protein kinase inhibitor-induced inflammatory alopecia in woman with ovarian cancer. J Drugs Dermatol. 2024;23(4):e102–3. 10.36849/JDD.7802. Describes a rare inflammatory pattern of MEK inhibitor-induced alopecia, contributing to the differential diagnosis of drug-related hair loss 10.36849/JDD.7802 38564383

[39] Oh DY, Bang YJ. HER2-targeted therapies – a role beyond breast cancer. Nat Rev Clin Oncol. 2020;17(1):33–48. 10.1038/s41571-019-0268-3. 31548601 10.1038/s41571-019-0268-3

[40] Dumontet C, Reichert JM, Senter PD, Lambert JM, Beck A. Antibody-drug conjugates come of age in oncology. Nat Rev Drug Discov. 2023;22(8):641–61. 10.1038/s41573-023-00709-2. (Offers a review of antibody-drug conjugates, linking new HER2 and Trop-2 therapies to alopecia via cytotoxic payload mechanisms). 37308581 10.1038/s41573-023-00709-2

[41] Böhme C. Anti-HER2 therapy: how to use Herceptin in clinical practice. Eur J Oncol Nurs. 2000;4(Sa):30–6. 10.1054/ejon.2000.0071. 12849615 10.1054/ejon.2000.0071

[42] Modi S, Saura C, Yamashita T, et al. Trastuzumab deruxtecan in previously treated HER2-positive breast cancer. N Engl J Med. 2020;382(7):610–21. 10.1056/NEJMoa1914510. 31825192 10.1056/NEJMoa1914510 PMC7458671

[43] Cortés J, Kim SB, Chung WP, et al. Trastuzumab deruxtecan versus trastuzumab emtansine for breast cancer. N Engl J Med. 2022;386(12):1143–54. 10.1056/NEJMoa2115022. 35320644 10.1056/NEJMoa2115022

[44] Hurvitz SA, Hegg R, Chung WP, et al. Trastuzumab deruxtecan versus trastuzumab emtansine in patients with HER2-positive metastatic breast cancer: updated results from DESTINY-Breast03, a randomised, open-label, phase 3 trial. Lancet. 2023;401(10371):105–17. 10.1016/S0140-6736(22)02420-5. (Updated phase 3 trial results showing alopecia incidence from trastuzumab deruxtecan; relevant for informing patients about hair loss risks). 36495879 10.1016/S0140-6736(22)02420-5

[45] Bardia A, Hurvitz SA, Tolaney SM, et al. Sacituzumab govitecan in metastatic triple-negative breast cancer. N Engl J Med. 2021;384(16):1529–41. 10.1056/NEJMoa2028485. 33882206 10.1056/NEJMoa2028485

[46] Spring LM, Tolaney SM, Fell G, et al. Response-guided neoadjuvant sacituzumab govitecan for localized triple-negative breast cancer: results from the NeoSTAR trial. Ann Oncol. 2024;35(3):293–301. 10.1016/j.annonc.2023.11.018. (Recent trial highlights high alopecia incidence with sacituzumab govitecan, important for treatment planning and counseling). 38092228 10.1016/j.annonc.2023.11.018

[47] Muntyanu A, Netchiporouk E, Gerstein W, Gniadecki R, Litvinov IV. Cutaneous immune-related adverse events (irAEs) to immune checkpoint inhibitors: a dermatology perspective on management. J Cutan Med Surg. 2021;25(1):59–76. 10.1177/1203475420943260. 32746624 10.1177/1203475420943260

[48] Jaber SH, Cowen EW, Haworth LR, et al. Skin reactions in a subset of patients with stage IV melanoma treated with anti-cytotoxic T-lymphocyte antigen 4 monoclonal antibody as a single agent. Arch Dermatol. 2006;142(2):166–72. 10.1001/archderm.142.2.166. 16490844 10.1001/archderm.142.2.166

[49] Assi H, Wilson KS. Immune toxicities and long remission duration after ipilimumab therapy for metastatic melanoma: two illustrative cases. Curr Oncol. 2013;20(2):165. 10.3747/co.20.1265. 10.3747/co.20.1265 PMC3615868 23559884

[50] Pearson DR, Lewis K, Alkousakis T. Remote-onset alopecia areata attributed to ipilimumab. Cutis. 2019;104(6):E25–7. 31939941

[51] Belum VR, Benhuri B, Postow MA, et al. Characterisation and management of dermatologic adverse events to agents targeting the PD-1 receptor. Eur J Cancer. 2016;60:12–25. 10.1016/j.ejca.2016.02.010. 27043866 10.1016/j.ejca.2016.02.010 PMC4998047

[52] Ito T. Immune checkpoint inhibitor-associated alopecia areata. Br J Dermatol. 2017;176(6):1444–5. 10.1111/bjd.15591. 28581240 10.1111/bjd.15591

[53] Sibaud V. Dermatologic reactions to immune checkpoint inhibitors: skin toxicities and immunotherapy. Am J Clin Dermatol. 2018;19(3):345–61. 10.1007/s40257-017-0336-3. 29256113 10.1007/s40257-017-0336-3

[54] Zarbo A, Belum VR, Sibaud V, et al. Immune-related alopecia (areata and universalis) in cancer patients receiving immune checkpoint inhibitors. Br J Dermatol. 2017;176(6):1649–52. 10.1111/bjd.15237. 27943234 10.1111/bjd.15237 PMC5459625

[55] Antoury L, Maloney NJ, Bach DQ, Goh C, Cheng K. Alopecia areata as an immune-related adverse event of immune checkpoint inhibitors: a review. Dermatol Ther. 2020;33(6): e14171. 10.1111/dth.14171. 32799412 10.1111/dth.14171

[56] Kruse M, Abraham J. Management of chemotherapy-induced alopecia with scalp cooling. J Oncol Pract. 2018;14(3):149–54. 10.1200/JOP.17.00038. 29529389 10.1200/JOP.17.00038

[57] Shin H, Jo SJ, Kim DH, Kwon O, Myung SK. Efficacy of interventions for prevention of chemotherapy-induced alopecia: a systematic review and meta-analysis. Int J Cancer. 2015;136(5):E442–54. 10.1002/ijc.29115. 25081068 10.1002/ijc.29115

[58] Villarreal-Garza C, Mesa-Chavez F, Garza-Ledezma MRA, et al. Impact of chemotherapy regimen and sequence on the effectiveness of scalp cooling for alopecia prevention. Breast Cancer Res Treat. 2021;185(2):453–8. 10.1007/s10549-020-05968-w. 33125621 10.1007/s10549-020-05968-w

[59] Suchonwanit P, Thammarucha S, Leerunyakul K. Minoxidil and its use in hair disorders: a review. Drug Des Devel Ther. 2019. 10.2147/DDDT.S214907. 31496654 10.2147/DDDT.S214907 PMC6691938

[60] Patel P, KD NT. Minoxidil. In: *StatPearls*. Treasure Island (FL): StatPearls Publishing; 2025 Jan–. Available from: https://www.ncbi.nlm.nih.gov/books/NBK482378/

[61] Duvic M, Lemak NA, Valero V, Hymes SR, Farmer KL, Hortobagyi GN, et al. A randomized trial of minoxidil in chemotherapy-induced alopecia. J Am Acad Dermatol. 1996;35(1):74–8. 10.1016/S0190-9622(96)90500-9. 8682968 10.1016/S0190-9622(96)90500-9

[62] Freites-Martinez A, Chan D, Sibaud V, et al. Assessment of quality of life and treatment outcomes of patients with persistent postchemotherapy alopecia. JAMA Dermatol. 2019;155(6):724–8. 10.1001/jamadermatol.2018.5071. 30840033 10.1001/jamadermatol.2018.5071 PMC6563563

[63] Yang X, Thai KE. Treatment of permanent chemotherapy-induced alopecia with low dose oral minoxidil. Australas J Dermatol. 2016;57(4):e130–2. 10.1111/ajd.12350. 25966934 10.1111/ajd.12350

[64] Lyakhovitsky A, Segal O, Maly A, Zlotogorski A, Barzilai A. Permanent chemotherapy-induced alopecia after hematopoietic stem cell transplantation treated with low-dose oral minoxidil. JAAD Case Rep. 2022;22:64–7. 35321258 10.1016/j.jdcr.2022.01.035 PMC8935347

[65] Kang J, Lee JW, Kwon O. Efficacy of low-dose oral minoxidil in the management of anticancer therapy-induced alopecia in patients with breast cancer: a retrospective cohort study. J Am Acad Dermatol. 2023;88(5):1170–3. 10.1016/j.jaad.2022.12.005. (Provides real-world evidence on the use of oral minoxidil in cancer-related alopecia, which is especially valuable given limited data). 36526083 10.1016/j.jaad.2022.12.005

[66] Minta A, Park C, Rose L, Trovato S, Dulmage B. Retrospective review of oral and topical minoxidil for cancer treatment-induced hair loss. Arch Dermatol Res. 2023;315(9):2613–5. 10.1007/s00403-023-02660-z. (Compares oral vs. topical minoxidil, supporting its emerging role in CRA management and patient-centered decision making). 37421421 10.1007/s00403-023-02660-z

[67] Wikramanayake TC, Haberland NI, Akhundlu A, Laboy Nieves A, Miteva M. Prevention and treatment of chemotherapy-induced alopecia: what is available and what is coming? Curr Oncol. 2023;30(4):3609–26. 10.3390/curroncol30040275. (Comprehensive review of current and pipeline therapies for CIA; provides a summary of what is available and what is next). 37185388 10.3390/curroncol30040275 PMC10137043

[68] Wang C, Du Y, Bi L, Lin X, Zhao M, Fan W. The efficacy and safety of oral and topical spironolactone in androgenetic alopecia treatment: a systematic review. Clin Cosmet Investig Dermatol. 2023;16:603–12. 10.2147/CCID.S398950. (While focused on androgenetic alopecia, it evaluates spironolactone’s potential, indirectly relevant to CRA management in hormone-sensitive cancers). 36923692 10.2147/CCID.S398950 PMC10010138

[69] Aguilar Medina DA, Cazarín J, Magaña M. Spironolactone in dermatology. Dermatol Ther. 2022;35(5): e15321. 10.1111/dth.15321. 35038224 10.1111/dth.15321

[70] Sharma A, Goren A, Dhurat R, et al. Tretinoin enhances minoxidil response in androgenetic alopecia patients by upregulating follicular sulfotransferase enzymes. Dermatol Ther. 2019;32(3): e12915. 10.1111/dth.12915. 30974011 10.1111/dth.12915

[71] Smith S, Fagien S, Whitcup SM, et al. Eyelash growth in subjects treated with bimatoprost: a multicenter, randomized, double-masked, vehicle-controlled, parallel-group study. J Am Acad Dermatol. 2012;66(5):801–6. 10.1016/j.jaad.2011.06.005. 21899919 10.1016/j.jaad.2011.06.005

[72] Ahluwalia GS. Safety and efficacy of bimatoprost solution 0.03% topical application in patients with chemotherapy-induced eyelash loss. J Investig Dermatol Symp Proc. 2013;16(1):S73-6. 10.1038/jidsymp.2013.30. 24326568 10.1038/jidsymp.2013.30

[73] Glaser DA, Hossain P, Perkins W, et al. Long-term safety and efficacy of bimatoprost solution 0.03% application to the eyelid margin for the treatment of idiopathic and chemotherapy-induced eyelash hypotrichosis: a randomized controlled trial. Br J Dermatol. 2015;172(5):1384–94. 10.1111/bjd.13443. 25296533 10.1111/bjd.13443 PMC4832276

[74] Baiyasi M, St Claire K, Hengy M, et al. Eyelash serums: a comprehensive review. J Cosmet Dermatol. 2024;23(7):2328–44. 10.1111/jocd.16278. (Evaluates prostaglandin-based eyelash serums; increasingly relevant for patients experiencing chemotherapy-induced eyelash loss). 10.1111/jocd.16278 38475901

[75] Lodewijckx J, Robijns J, Claes M, et al. The use of photobiomodulation therapy for the management of chemotherapy-induced alopecia: a randomized, controlled trial (HAIRLASER trial). Support Care Cancer. 2023;31(5):269. 10.1007/s00520-023-07743-1. (Presents randomized trial data on photobiomodulation therapy a promising adjunct or alternative for CRA). 37060420 10.1007/s00520-023-07743-1

[76] Tkachenko E, Okhovat JP, Manjaly P, et al. Complementary and alternative medicine for alopecia areata: a systematic review. J Am Acad Dermatol. 2023;88(1):131–43. 10.1016/j.jaad.2019.12.027. (While focused on alopecia areata, this systematic review includes alternative therapies applicable to patient preferences in CRA). 31870916 10.1016/j.jaad.2019.12.027

[77] Hosking AM, Juhasz M, Atanaskova Mesinkovska N. Complementary and alternative treatments for alopecia: a comprehensive review. Skin Appendage Disord. 2019;5(2):72–89. 10.1159/000492035. 30815439 10.1159/000492035 PMC6388561

[78] Ablon G, Dayan S. A randomized, double-blind, placebo-controlled, multi-center, extension trial evaluating the efficacy of a new oral supplement in women with self-perceived thinning hair. J Clin Aesthet Dermatol. 2015;8(12):15–21. 26705444 PMC4689507

[79] Rizer RL, Stephens TJ, Herndon JH, et al. A marine protein-based dietary supplement for subclinical hair thinning/loss: results of a multisite, double-blind, placebo-controlled clinical trial. Int J Trichology. 2015;7(4):156–66. 10.4103/0974-7753.171573. 26903744 10.4103/0974-7753.171573 PMC4738482

[80] Ablon G. A 3-month, randomized, double-blind, placebo-controlled study evaluating the ability of an extra-strength marine protein supplement to promote hair growth and decrease shedding in women with self-perceived thinning hair. Dermatol Res Pract. 2015;2015: 841570. 10.1155/2015/841570. 25883641 10.1155/2015/841570 PMC4389977

[81] Ablon G, Kogan S. A six-month, randomized, double-blind, placebo-controlled study evaluating the safety and efficacy of a nutraceutical supplement for promoting hair growth in women with self-perceived thinning hair. J Drugs Dermatol. 2018;17(5):558–65. 29742189

[82] Ablon G, Kogan S. A randomized, double-blind, placebo-controlled study of a nutraceutical supplement for promoting hair growth in perimenopausal, menopausal, and postmenopausal women with thinning hair. J Drugs Dermatol. 2021;20(1):55–61. 10.36849/JDD.5701. 33400421 10.36849/JDD.5701

